# Supplementary material for: Crude Heparin Preparations Unveil the Presence of Structurally Diverse Oversulfated Contaminants
Source: Molecules. 2019 Aug 17;24(16):2988. doi: 10.3390/molecules24162988 (PMC6721129; doi:10.3390/molecules24162988)
Supplement: Supplementary file 1 [file molecules-24-02988-s001.pdf]

SUPPLEMENTARY MATERIALS

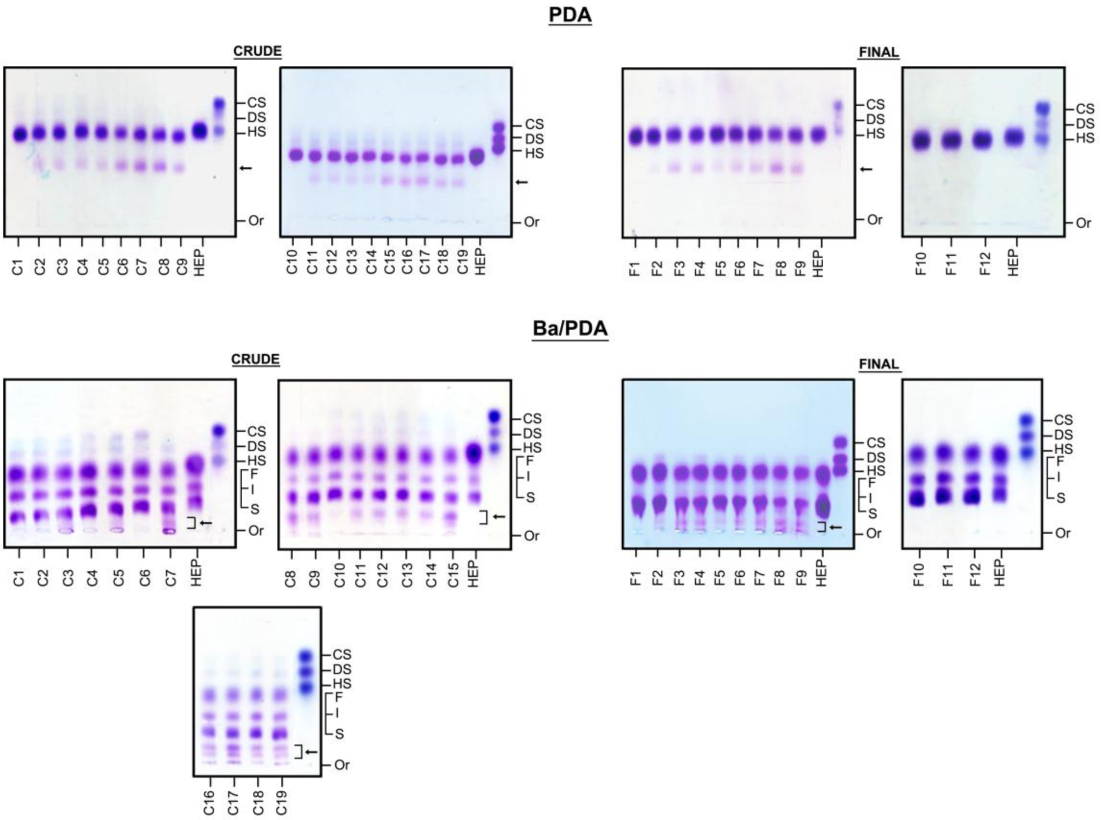

Figure S1: Identification of contaminants in crude and final heparins by agarose gel electrophoresis. Aliquots (5 μL, 5-10 μg) of heparin samples were applied to 0.55% agarose gel and submitted to electrophoresis in 0.05M PDA or Ba/PDA discontinuous system as described in Methods. The arrow shows the presence of slow migrating bands which are not present in standard heparin. In the discontinuous Ba/PDA buffer this is most evident due to a clear separation of slow migrating bands. The figure also shows differences in the amounts of these contaminants among both the crude and final product heparin.

C1 to C19: crude heparin samples; F1 to F12: final heparin samples; Hep: standard porcine heparin; CS/DS/HS: standard mixture of chondroitin sulfate (CS), dermatan sulfate (DS), and heparan sulfate (HS); S: heparin slow moving component, I: heparin intermediate moving component; F: heparin fast moving component.

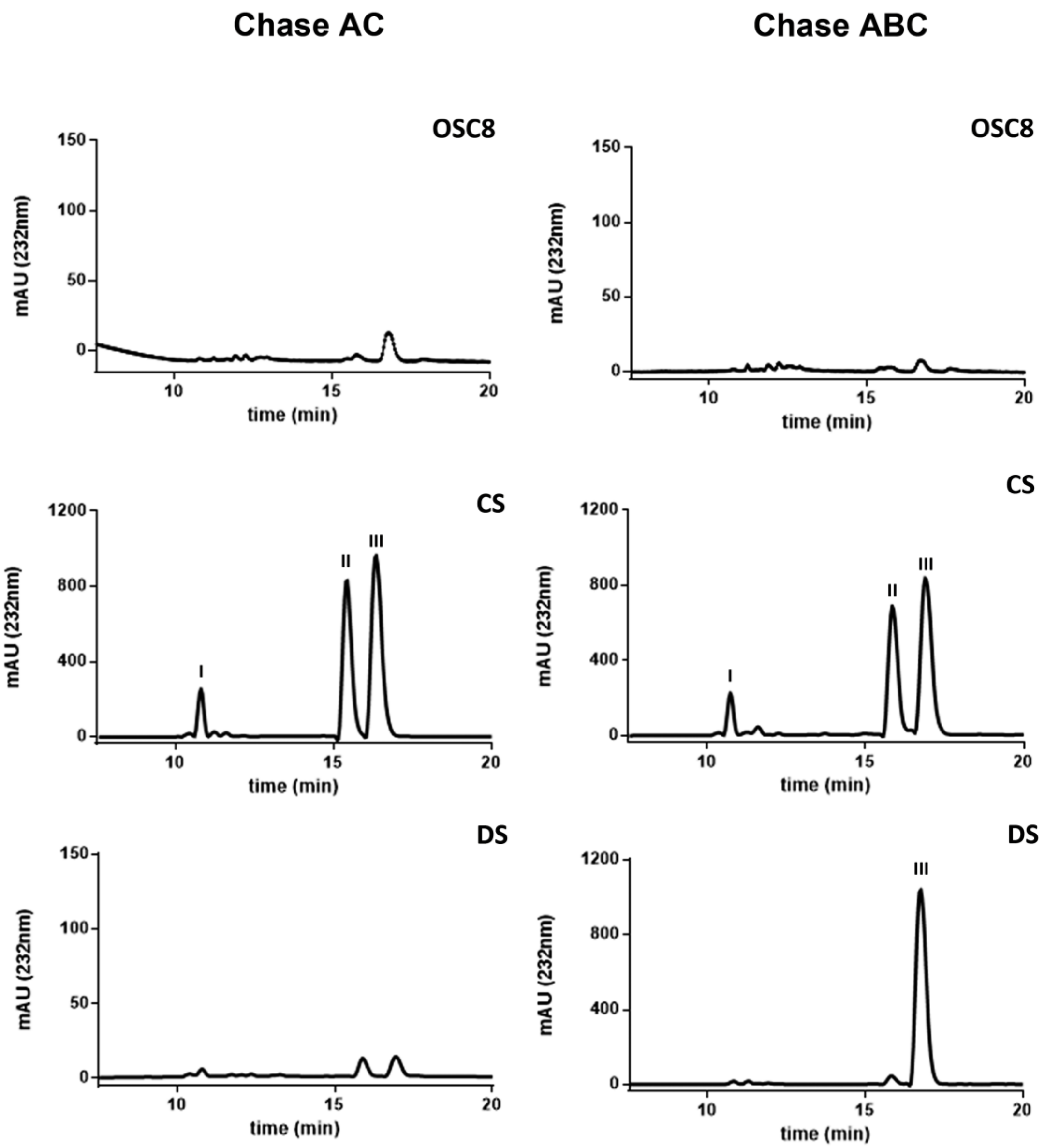

Figure S2: Analysis of the products formed after exhaustive degradation with chondroitinase AC and ABC by SAX-HPLC ion exchange chromatography. Samples of the isolated contaminant (OSCS) (100 μg) were incubated with 30 mU of the chondroitinase AC (from *Arthrobacter aurescens*) and ABC (from *Proteus vulgaris*) in 50mM sodium acetate buffer pH 8.0. The products were resolved in Zorbax SAX column with 0-1.5 M NaCl gradient with a flow of 1 mL/min for 30 min and the unsaturated products monitored by absorbance at 232 nm. Under conditions of exhaustive degradation (72h and 0.3 enzyme units) no product formation was observed, even though the polysaccharide is non-sulfated at position C-3 of the glucuronic. On the other hand, the enzymes act upon chondroitin sulfate (CS) and dermatan sulfate (DS) yielding the expected products. Chase AC: chondroitinse AC; Chase ABC: chondroitinase ABC; CS: Chondroitim sulfate; DS: dermatan sulfate; OSC8: sulfated contaminant isolated from crude heparin C8; Peak I: ΔDi0S (non-sulfated unsaturated disaccharide);

Peak II: ΔDi6S (6-sulfated unsaturated disaccharide); Peak III: ΔDi4S (4-sulfated unsaturated disaccharide).

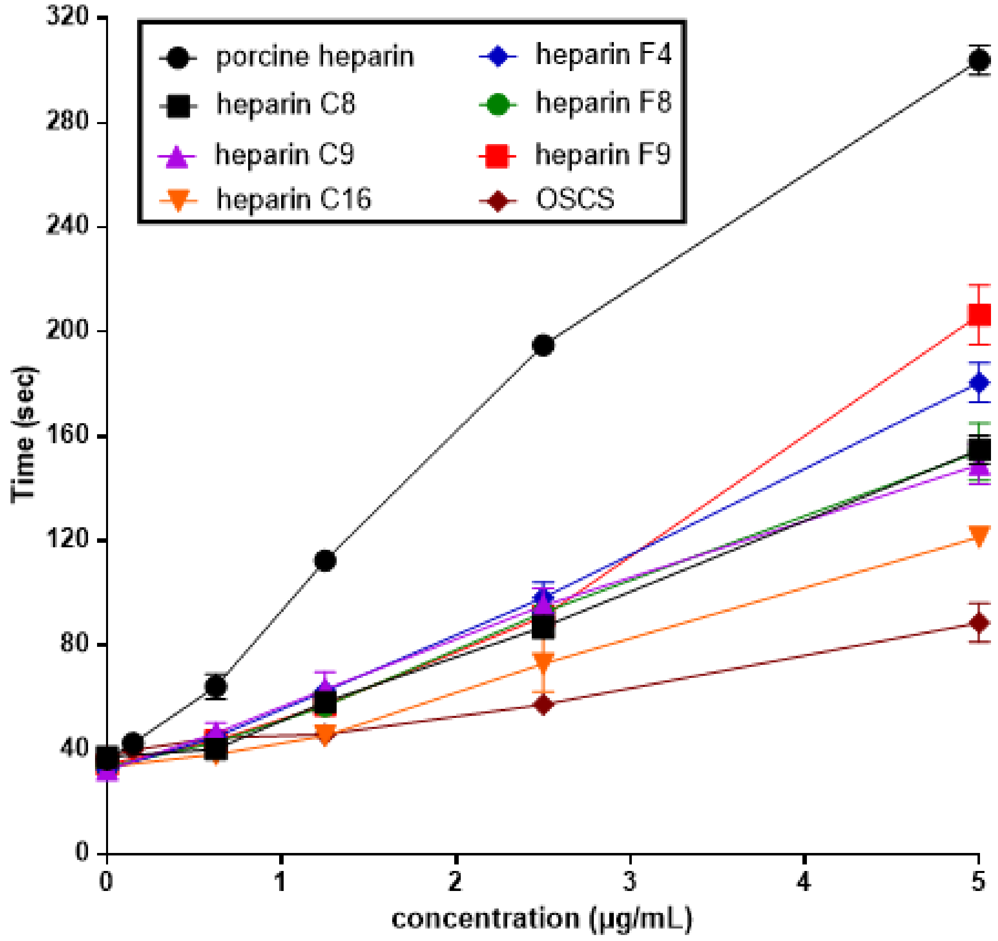

Figure S3. Comparative *in vitro* anticoagulant profile of heparin and crude and final product heparin samples in the APTT assay. From 0.625 μg/mL to up 5.0 μg/mL concentration, the contaminated heparins showed a significant decrease in the APTT compared to the standard heparin. The data represent the mean ± SEM of triplicates.

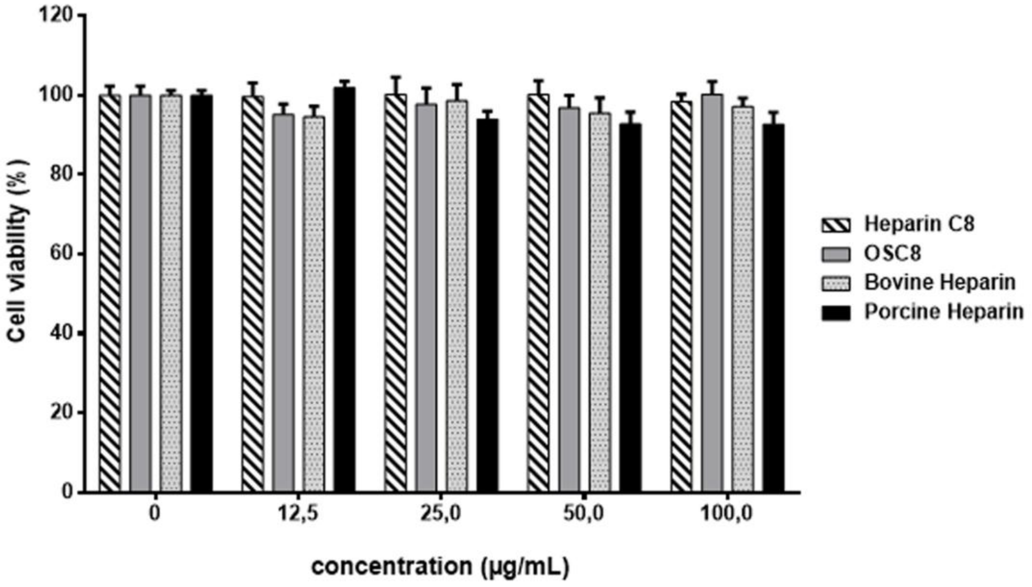

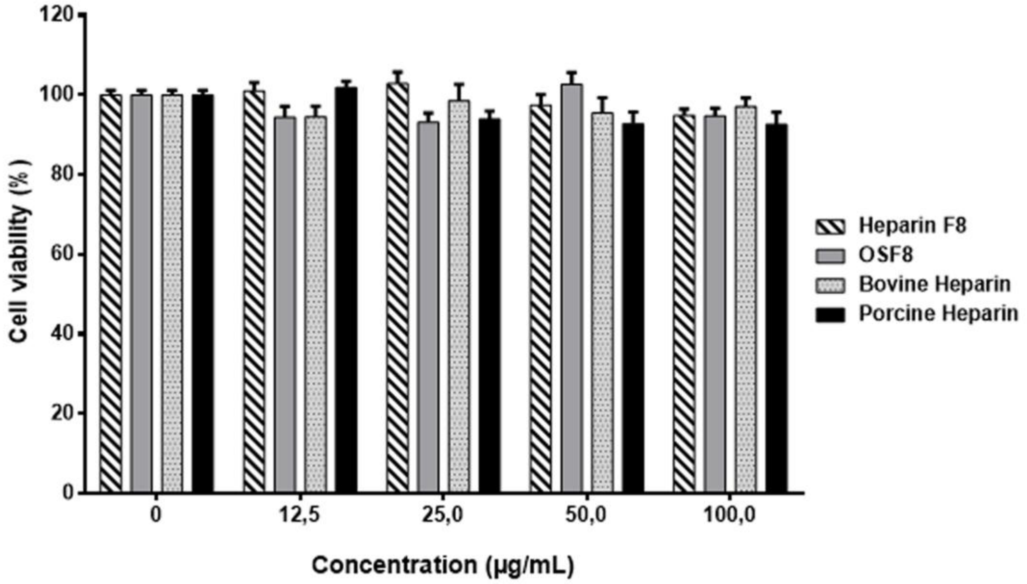

Figure S4: Effect of sulfated contaminants on the viability of endothelial cells. EC were plated in 96-well microplates (1 x 10^4 cells per well in enriched F12) and maintained for 24 h in an incubator at 37 °C in 2.5% CO_2 atmosphere. The cells were then exposed to different concentrations of the batches of heparins or isolated contaminants in 150 µL of enriched F12 for 24 h at 37 °C in 2.5% CO_2 atmosphere. Afterwards the medium was aspirated and 200 µL/well Alamar Blue® (0.1 mg/mL) solution prepared in serum-free culture medium added. The cells were then incubated for 4 h at 37 °C in an atmosphere of 2.5% CO_2. Next, 100µL of the medium were transferred to 96-well microplates and the reduction of the rezasurin salt in resorufin was measured by fluorescence analysis (λExc = 560nm; λEm = 590nm) in FlexStation[3] PlateReader, Molecular Devices. The results show no effect of the compounds in all tested concentrations. Heparin C8: crude

heparin C8; OSC8: sulfated contaminant purified from crude heparin C8; OSF8: sulfated contaminant purified from final heparin F8.

Table S1. Samples of crude and final product heparins used in this study (supplementary materials)

Crude heparin samples		Final heparin samples	
Sample	Batch	Sample	Batch
C1	CSP-609086	F1	1060-07-0002
C2	CSP-609087	F2	1060-07-0022
C3	CSP-709019	F3	1060-07-0023
C4	CSP-709020	F4	1060-07-0025
C5	CSP-709021	F5	1060-07-0026
C6	CSP-709024	F6	1060-07-0027
C7	CSP-709025	F7	1060-07-0028
C8	CSP-709026	F8	1060-07-0029
C9	CSP-709027	F9	1060-07-0030
C10	CSP-709028	F10	1060-05-0002
C11	CSP-709029	F11	1060-05-0023
C12	CSP-709030	F12	1060-05-0027
C13	CSP-709031		
C14	CSP-709032		
C15	CSP-709033		
C16	CSP-709034		
C17	CSP-709035		
C18	CSP-709036		
C19	CSP-709037		

Table S2. Percentage of contaminants in crude and final product heparins (supplementary materials)

Crude heparin samples	Heparin (%)	Contaminant (%)	Final heparin samples	Heparin (%)	Contaminant (%)
C1	100	-	F1	100	-
C2	92.2	7.8	F2	96.3	3.7
C3	92.4	7.6	F3	86.5	13.5
C4	90.2	9.8	F4	88.9	11.1
C5	91.1	8.9	F5	95.7	4.3
C6	76.2	23.8	F6	91.4	8.6
C7	73.7	26.3	F7	88.4	11.6
C8	71.9	28.1	F8	74.8	25.2
C9	82.0	18.0	F9	81.0	18.1
C10	100	-	F10	100	-
C11	87.3	12.7	F11	100	-
C12	87.1	12.9	F12	100	-
C13	89.5	10.5	Standard	100	-
C14	89.7	10.3			
C15	78.7	21.3			
C16	75.5	24.5			
C17	73.2	26.8			
C18	81.2	18.8			
C19	80.5	19.5			
